# Extending the Collection Duration of Breath Samples for Enteric Methane Emission Estimation Using the SF_6_ Tracer Technique

**DOI:** 10.3390/ani2020275

**Published:** 2012-06-08

**Authors:** César Pinares-Patiño, José Gere, Karen Williams, Roberto Gratton, Paula Juliarena, German Molano, Sarah MacLean, Edgar Sandoval, Grant Taylor, John Koolaard

**Affiliations:** 1 Grasslands Research Centre, AgResearch Limited, Palmerston North 4442, New Zealand; E-Mails: german.molano@agresearch.co.nz (G.M.); sarah.maclean@agresearch.co.nz (S.M.); edgar.sandoval@agresearch.co.nz (E.S.); grant.taylor@agresearch.co.nz (G.T.); john.koolaard@agresearch.co.nz (J.K.); 2 Facultad de Ciencias Exactas, Universidad Nacional del Centro de la Provincia de Buenos Aires, Pinto 399, Tandil 7000, Buenos Aires, Argentina; E-Mails: jgere@exa.unicen.edu.ar (J.G.); kwilliams@exa.unicen.edu.ar (K.W.); rgratton@rec.unicen.edu.ar (R.G.); pjuliarena@exa.unicen.edu.ar (P.J.)

**Keywords:** SF_6_ tracer, breath collection, collection duration, methane, cattle

## Abstract

**Simple Summary:**

Extended sample collection for the SF_6_ tracer technique is desirable for extensive grazing systems. Breath samples from eight cows were collected while lucerne silage was fed to achieve fixed intakes among the cows. Samples were collected over a 10-day period, using either apparatuses used in New Zealand (NZL) or Argentina (ARG), and either daily, over two consecutive 5-day periods or over a 10-day period (in duplicate). The NZL system had a greater sampling success and more consistent CH_4_ emission estimates than the ARG system, with no differences in mean emissions among sample collection periods. This study showed that extended sample collection is feasible, but definitive evaluation under grazing situation is required before a decision on recommendation can be made.

**Abstract:**

The daily sample collection protocol of the sulphur hexafluoride (SF_6_) tracer technique for the estimation of methane (CH_4_) emissions from ruminants may not be practical under extensive grazing systems. Here, under controlled conditions, we evaluated extended periods of sampling as an alternative to daily sample collections. Eight rumen-fistulated cows were housed and fed lucerne silage to achieve common daily feed intakes of 6.4 kg dry matter per cow. Following SF_6_ permeation tube dosing, eight sampling lines were fitted to the breath collection harness, so that a common gas mix was available to each line. Half of the lines collected samples into PVC yokes using a modified capillary system as commonly used in New Zealand (NZL), and half collected samples into stainless steel cylinders using a ball-bearing flow restrictor as used in Argentina (ARG), all within a 10-day time frame, either daily, across two consecutive 5-day periods or across one 10-day period (in duplicate). The NZL system had greater sampling success (97.3 *vs.* 79.5%) and yielded more consistent CH_4_ emission estimates than the ARG system. Emission estimates from NZL daily, NZL 5-day and NZL 10-day samplings were 114, 110 and 111 g d^−1^, respectively. Extended sample collection protocol may be feasible, but definitive evaluation of this alternative as well as sample collection systems is required under grazing situations before a decision on recommendation can be made.

## 1. Introduction

The sulphur hexafluoride (SF_6_) tracer technique [1] is currently the only method which enables the simultaneous estimation of methane (CH_4_) emissions from individual grazing animals in a large flock or herd [2]. This method employs a calibrated SF_6_ source (a permeation tube) pre-inserted into the reticulo-rumen of the participating animal and a light-weight “breath”-collection apparatus (evacuated PVC canister and capillary flow-restrictor) mounted on the animal [1]. The tracer technique has been widely used in New Zealand and overseas, and although there is evidence from controlled indoor experiments that the SF_6_ tracer technique exacerbates within- and between-animal variability of the estimated CH_4_ emissions [3], research in New Zealand [4,5] and overseas [1,6] comparing the tracer and respiration chamber techniques has shown average tracer estimates matching the average chamber values. 

Methane emission estimations using the SF_6_ tracer technique involve collections of 24-h integrated ‘breath’ samples over various time periods (usually 5−7 days). This original protocol [1] is followed in New Zealand, where evacuated canisters (made of PVC) and slightly modified capillary flow restrictors (a short capillary with a pressed section) are used. This modified sample collection apparatus is hereafter named New Zealand modified system (NZL system). In turn, researchers at the Universidad del Centro de la Provincial de Buenos Aires (UNICEN, Buenos Aires, Argentina) [7] have modified the original sample collection apparatus by using stainless steel sample collection cylinders and brass ball-bearing sample flow restrictors (hereafter named modified Argentinean system, ARG system). The Argentinean colleagues have proposed extended sample collection periods (over five or 10 days), as an alternative to the repeated 24-h integrated sample collections. Extended sample collection periods may be desirable in situations (e.g., resource-limited research institutions, extensive grazing systems, *etc.*) where daily sample collections may not be practical. 

The main objective of this study was to evaluate extended sample collection periods as an alternative to the daily sampling protocol of the SF_6_ tracer technique for CH_4_ emission estimation. A secondary objective was to compare the performance of the alternative sample collection apparatuses (ARG and NZL systems) for extended sample collection periods. For this purpose, breath samples from cattle were simultaneously collected using the ARG and NZL apparatuses, involving either daily, 5-day or 10-day sample collection periods. The study was conducted under controlled indoor conditions, hence it is preliminary and findings cannot be extrapolated to grazing situations. 

## 2. Materials and Methods

The study was conducted at the Ulyatt-Reid Large Animal Facility of AgResearch Grasslands Research Centre (Palmerston North, New Zealand). The study was approved by the Animal Ethics Committee of AgResearch Limited and animals were cared for with compliance with the Animal Welfare Act (1999). The cows used in this study were previously used in other studies involving the SF_6_ tracer technique.

### 2.1. Experimental Design

Eight rumen-fistulated non-lactating Friesian × Jersey dairy cows (524 ± 35.2 kg live weight) were used in the study. Animals were acclimatised to the experimental diet over 10 days while in a large pen (20 × 10 m), where they were group-fed. Then, the cows were transferred to individual stalls where they continued acclimatisation for three days. The housing environment had both natural and artificial ventilation. The natural ventilation was provided throughout by a perforated corrugated iron wall along the length of the building, whereas artificial ventilation was provided by eight centrifugal roof fans (Gamma Series CD568D, Fantech Pty. Lt., Victoria, Australia). The first three days in stalls were also used for acclimatisation of the animals to the ‘breath’ sampling apparatuses. The experiment was carried out over a 10-day period (days 1–10) when the CH_4_ emissions from all the individual animals were estimated by the SF_6_ tracer technique using both the ARG and NZL sample collection apparatuses, which involved three simultaneous sample collection periods: daily over 10 days (daily), across two consecutive 5-day periods (5-day) and across a 10-day period (10-day). 

### 2.2. Feed and Feed Intake

The animals were fed on a commercial molassed lucerne silage diet, which was sourced from a single batch and had a dry matter (DM) content of 446 g/kg, and crude protein, neutral detergent fiber and soluble sugars contents of 222, 466 and 39 g kg^−1^ DM, respectively, whereas the metabolisable energy content was 9.2 MJ kg^−1^ DM. Feed on offer was set at 1.2 times the mean cow maintenance energy requirements [8]. Thus, throughout the study all cows were offered the same amount of feed, which was delivered in two equal meals at 08:30 and 16:00 h. All the cows consumed all their offered feed within 30 min of delivery, achieving a DM intake (DMI) of 6.40 kg d^−1^. Drinking water was provided *ad libitum*. 

### 2.3. The CH_4_ Measurement (Days 1–10)

The SF_6_ permeation tubes used in the experiment were selected from a larger number (n = 20) to minimise the range of permeation rates. The selected tubes had an average permeation rate (PR) of SF_6_ of 4.253 ± 0.116 mg d^−1^ (range 4.030–4.384 mg d^−1^) and at the time of their deployment in the reticulum (*per os* dosing), were 125 days old and breath samples were collected after 10 days of tube deployment. The permeation tubes remained in the cows throughout the study and were retrieved (via ruminal fistula) after 28 days of deployment. 

**Figure 1:**
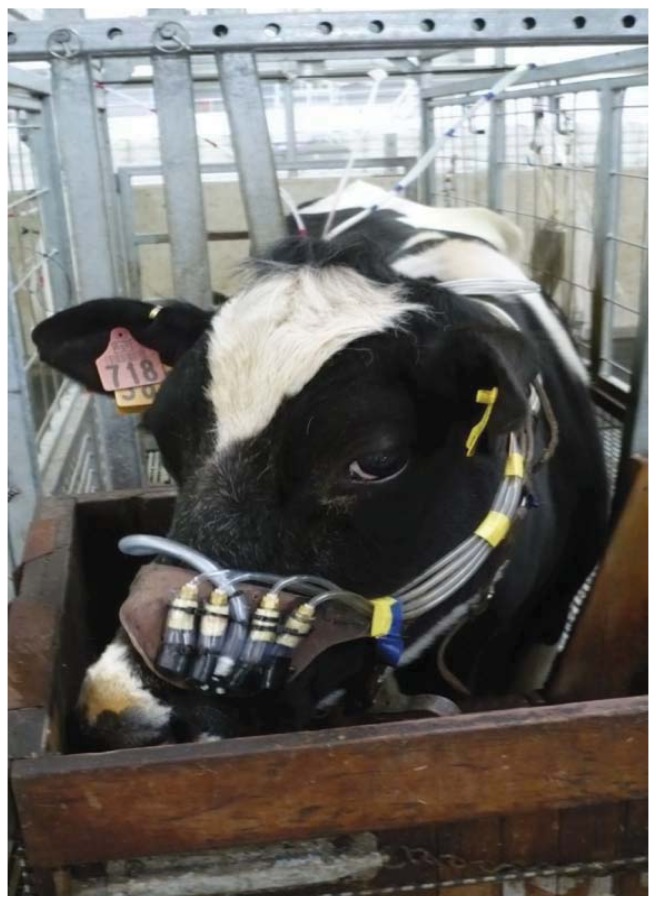
Each cow harness was fitted with eight breath sample lines to allow a similar source of ‘breath’ sample just above the animal’s nostrils. Half of the sample lines were allocated to the Argentinean modified system (ARG) and another half to the New Zealand modified system (NZL). Sampling lines allocated to the NZL system were enclosed within polyethylene tubing having a common sample inlet (inverted ‘Y’) and placed at the mid-point between two ARG sample lines. Each of the ball-bearing flow restrictors for the ARG system (the sample inlet) was covered by water-proof polyester material. All the samples lines were extended to the back sides of the animal crate.

**Figure 2:**
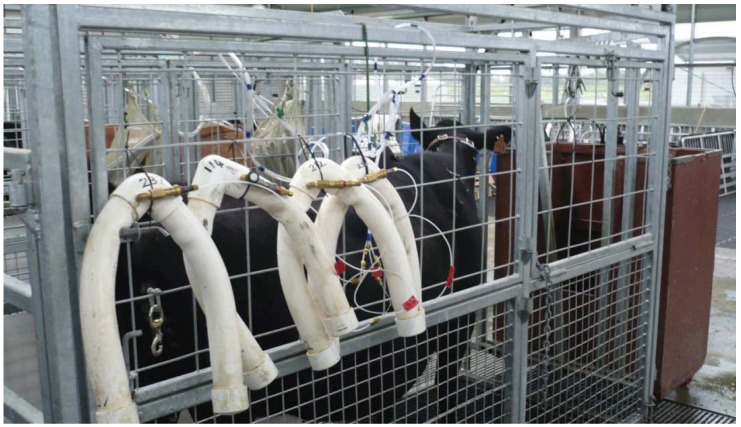
The sample collection canisters were hung at the rear sides of the animal crates. The PVC ‘yokes’ (NZL system) were located at the right side, whereas the stainless steel canisters (ARG system) were located at the left side. Sample lines were out of reach of the animal.

**Figure 3:**
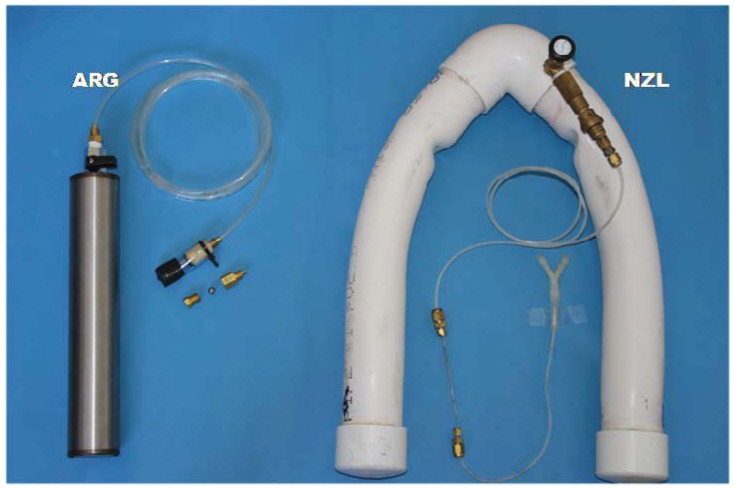
The ARG and NZL sample collection apparatuses. The ARG system involves a stainless steel cylinder (0.5 L) and a ball-bearing sample flow restrictor. The NZL system involves PVC ‘yoke’ (2.5 L) and a short capillary tube as sample flow restrictor.

Eight sample lines were fitted to the cow harness. All had a similar source of ‘breath’ sample just above the nostrils of each participating animal. Half of the sample lines were allocated to the NZL system and another half to the ARG system (Figure 1). All the sample lines were extended (5 m length) to the back sides of the crates out of reach of the animal, where the collection canisters were hung (Figure 2). The extension lines for the ARG system were 1/4" o.d. polyethylene tubing, whereas those for the NZL system were 1/8" o.d. nylon tubing. The ARG system used stainless steel cylinders (0.5 L volume) as sample collection devices with sample flow restricted by a steel ball-bearing [7]. Flow restriction was achieved by pressing a stainless steel ball (8 mm diameter) housed in a brass female thread socket (Part IR8GH, Casucci Automatizacion S.A., Buenos Aires, Argentina) by means of a 1/8" brass bolt (Part A-TX8G-NPT, Casucci Automatizacion S.A., Buenos Aires, Argentina). Each flow restrictor for the ARG system was housed into a 5 cm polyethylene tubing (12 mm i.d.), the end of which was covered by a polyester fabric (camping tent material) aimed at protecting the flow restrictor from water blockage (Figure 3). 

The NZL system used PVC yoke-shape collection devices (2.5 L volume) with sample flow restricted by a capillary system (Figure 3), which was located approximately 30 cm from the sample inlet. The capillary system was slightly modified from the original recommendation [9] by pressing a small section (4 mm) of a short length (10 cm) of stainless steel capillary tubing (1/16" o.d., 0.003"−0.005" i.d.; Alltech®) until the desired flow was achieved. All four sample inlets for the NZL systems were also housed in polyethylene tubing, to which end an inverted ‘Y’ union was attached (Figure 3). Both the stainless steel and PVC canisters were pre-evacuated (<3 kPa pressure) previous to each sample collection. The sample flow restrictors (ball-bearing and capillary) used in this study were calibrated in the laboratory to allow appropriate sample collections for the desired collection periods, *i.e.*, a final pressure of 60–65 kPa at the end of the sample collection periods. 

Within each system (ARG and NZL) and within a 10-day time frame, common-source breath samples were collected either daily (ARG daily and NZL daily), across two consecutive 5-day periods (ARG 1–5, ARG 6–10, NZL 1–5, NZL 6–10; hereafter named ARG 5-day and NZL 5-day) or across one 10-day period (ARG 10-day, NZL 10-day). Both the ARG 10-day and NZL 10-day collections were conducted in duplicate. If necessary (e.g., when flow restrictors were blocked), some sample collection lines (ARG daily, NZL daily, ARG 1–5 and NZL 1–5) were replaced by new units. Within each sample collection period, samples were declared appropriate for analyses (successful sampling) when the collection canister had the desired final residual pressure (60–65 kPa). Otherwise, samples with very low pressure (0−30 kPa) or near-atmospheric pressure (80−100 kPa) at the end of the scheduled collection period were deemed inappropriate for analyses.

In addition to the ‘breath’ samples, background air samples were collected daily, over two consecutive 5-day periods or across a 10-day period. These samples were collected in duplicate (exception was for the 10-day period) 10 m from each other (the duplicates) and at 6 m from the stalls, and facing the direction of the natural ventilation. Background samples were collected using both the ARG and NZL systems. Immediately after the collection periods, breath and background samples were analysed for concentrations of CH_4_ (ppm, parts per million by volume) and SF_6_ (ppt, parts per trillion by volume) by gas chromatography (GC2010, Shimadzu, Tokio, Japan) as described by Pinares-Patiño *et al*. [3].

### 2.4. Calculations and Statistical Analysis

Estimates of emissions of CH_4_ over the sample collection periods (daily, 5-days and 10-days) were calculated from the specific PR of SF_6_ and the CH_4_/SF_6_ ratio of mixing ratios (volume/volume) in breath samples, after correction for background gas concentrations [9]. For this purpose PR of SF_6_ were expressed per day, consequently the emissions estimates from daily, 5-day and 10-day sample collections corresponded to daily emissions. Within systems (ARG *vs.* NZL), data for each cow was averaged within each sample collection duration. Estimates of CH_4_ emissions were expressed on absolute basis (g d^−1^; 1 g CH_4_ = 1.4 L CH_4_) only. There was no need for expression of emissions on feed intake basis, as all the animals had common feed intakes throughout the study. 

Data for CH_4_ emission estimates were analysed in GenStat [10] with the residual maximum likelihood method (REML) to estimate the between-cow and within-cow variance. The CH_4_ emission estimates were those derived from each sampling period (daily, 5-day and 10-day) within each sampling system (ARG and NZL). Paired t-tests using differences between cow means were used for pairwise comparisons of CH_4_ emissions between sample collection periods (daily, 5-day, and 10-day) within sampling systems (ARG and NZL). Collection system (ARG *vs.* NZL) effects on mean CH_4_ emissions were compared for sample collections of similar duration only. Pearson coefficients of correlation between the CH_4_ emission estimates within the sample collection systems were also calculated. Within each sample collection duration (within each system), data for each cow were averaged and the paired t-tests and coefficients of correlation were conducted on these within-cow averaged data. 

## 3. Results and Discussion

### 3.1. Sample Collection Success

Sample collections failed to achieve 100% success (Table 1). Overall, the ARG system had a greater number of sample losses than the NZL system (23 *vs.* 4). For example, for the 10-day sample collection duration, collections were fully successful for the NZL system, whereas the ARG system had only 9 out 16 acceptable samples, with one cow losing both the duplicate samples. In all cases the sample losses for the NZL system were due to blockage of the sample flow restrictors by water. In addition to blockage of flow restrictors by water, sample losses within the ARG system were due to excess vacuum loss from sample containers, a fact most likely resulting from the imposed experimental conditions (extension of sample line). During the fourth day of sampling, the eight daily samples of the ARG system were lost because the cylinders were not switched on (human error). The number of lost samples for the ARG system due to water blockage, excess vacuum loss and human error were 5, 10 and 8, respectively.

**Table 1:** Number of possible (maximum number) and successful samples obtained using the Argentinean modified system (ARG) and New Zealand modified system (NZL) sample collection apparatuses, each involving three simultaneous sample collection periods (daily, 5-day and 10-day).

	Daily	5-Day			10-Day	Total samples
	all	Days 1–5	Days 6–10	all	all	
Maximum	80	8	8	16	16	112
ARG successful	67	7	6	13	9	89
NZL successful	78	7	8	15	16	108

In this study, blockage of the sample flow restrictor with water occurred with both systems, but it was slightly more for the ARG system than for the NZL system. In the ARG system, the location of the flow restrictor with respect to the point source of sample (animal’s nostrils) could have had a major influence in blockages. In contrast to the NZL system where the flow restrictor (a capillary) was located away from the sample inlet, the flow restrictor in the ARG system was at the sampling point, *i.e.*, the flow restrictor constituted the inlet of the sample. Thus, the ARG system was more at risk of blockage by drinking water reaching the sample entrance. Most importantly, water blockage in the ARG system seemed to be triggered by condensation of breath moisture into the metal components of the flow restriction assembly. The ARG system uses a flow restrictor that is an assembly of a brass body housing a stainless steel ball (Figure 3). 

Excess loss of vacuum from containers (*i.e.*, residual pressure in container equal to atmosphere) in the ARG system occurred in the extended sample collection periods (mostly the 10-day sampling), and this was despite the proper calibration of the desired flow rate of the sample. The likely reason for this phenomenon was the location of the flow restrictor with respect to the sample collection container. In fact, pressure inside the 5 m flexible tube (1/4'' o.d.) was sub-atmospheric and hence prone to air leakage from outside. Leakage may have been facilitated by twisting of the sample line and torsion of line joins due to animal movements. Small leakage events may have become significant over the extended sample collection period and hence contributed to exhaustion of vacuum. In addition, physical contacts of the flow restrictors (close to animal nostrils) to the feeding bins (during feeding times) may have gradually loosened the flow restriction assembly. In contrast, in the NZL system the sample restrictor was well protected and away from the nostrils of the animals and the smaller diameter tube extension was well protected from leakages. The ARG and NZL systems fundamentally differ in the nature of their sample flow restrictors and findings from this study cannot be extrapolated to grazing conditions, especially when sample collection duration is extended.

### 3.2. Mean Estimates of Methane Emission and Between-Animal and Within-Animal Variability of Estimates

The mean concentrations (by volume) of CH_4_ and SF_6_ in the background air samples (Table 2) were within the range reported in the literature [11], suggesting that the building was appropriately ventilated. The mean concentrations (by volume) of CH_4_ and SF_6_ in breath samples found in this study were 57 ppm and 240 ppt, respectively.

**Table 2:** Mean (±standard deviation) concentrations (by volume) of methane (CH_4_, ppm) and sulphur hexafluoride (SF_6_, ppt) in the background air samples collected using the ARG and NZL sample collection apparatuses involving three simultaneous sample collection periods (daily, 5-day and 10-day). There was only one background sample for the 10-day sample duration.

	Daily		5-Day		10-Day	
	CH_4_	SF_6_	CH_4_	SF_6_	CH_4_	SF_6_
ARG	3.07 ± 0.71	11.56 ± 2.91	3.72 ± 0.62	12.63 ± 0.45	2.69	10.40
NZL	3.59 ± 0.56	12.49 ± 1.95	3.44 ± 0.27	13.26 ± 0.88	3.38	12.81

Within the ARG sample collection system, the mean emission estimate from daily samplings was significantly higher (*P* < 0.05) than the estimates from the extended sample collections (5-day and 10-day) (Table 3), whereas the extended sample collections (5-day and 10-day) did not differ from each other in their emission estimates (*P* > 0.05). In contrast, for the NZL system, the sample collections did not differ (*P* > 0.05) in their CH_4_ emission estimates (Table 3). No differences (*P* > 0.05) were found between ARG and NZL sample collection systems for their CH_4_ emission estimates when compared within particular collection periods, with the exception that the ARG 5-day estimate was smaller (*P* < 0.05) than that for NZL 5-day. The mean CH_4_ emission estimates for the ARG and NZL systems were 106 ± 10.7 and 111 ± 13.2 g d^−1^, respectively. These mean emission estimates equalled CH_4_ yields of 16.5 ± 1.7 and 17.4 ± 2.0 g kg^−1^ DMI for the ARG and NZL systems, respectively; values similar to that reported (17.7 g kg^−1^ DMI) from a previous study using similar feed [12]. Dry matter intake (6.40 kg d^−1^) was common for all the cows throughout the experiment. 

**Table 3:** Mean (±standard deviation) estimates of methane emission and between-animal and within-animal variances for methane emissions using the ARG and NZL sample collection apparatuses involving three simultaneous sample collection periods (daily, 5-day and 10-day).

	ARG system			NZL system		
	Daily (1–10)	5-day (1–5 and 6–10)	10-day (1–10)	Daily (1–10)	5-day (1–5 and 6–10)	10-day (1–10)
Mean emission (g d^−1^)	113.8 ± 7.4 ^a^	102.1 ± 9.1 ^b^	104.1 ± 12.5 ^b^	114.2 ± 13.0 ^a^	110.0 ± 12.8 ^a^	111.3 ± 13.5 ^a^
Between-animal variance	39.1	n.d.	n.d.	162.1	n.d.	172.8
Within-animal variance	136.1	81.7	132.5	78.5	188.8	20.7

Comparison between sample collection periods within systems: means within the ARG or the NZL system with different superscript letters (^a,b^) are significantly different (*P* < 0.05). Comparison between systems for similar collection periods (not shown in Table): only the means of the 5-day collections were significantly (*P* = 0.03) different (ARG 5-day < NZL 5-day). n.d.: not determined due to sample losses.

Table 3 also shows the between-animal and within-animal variance for the CH_4_ emission estimates. Sample collection losses for the 5-day sampling scheme within both the ARG and NZL systems (ARG 5-day and NZL 5-day) and also for the 10-day collection within the ARG system (ARG 10-day) precluded estimation of between-cow variances. The ARG daily sample collection (ARG daily) yielded the lowest between-cow variance. Both the NZL daily and the NZL 10-day samplings yielded similar between-animal variances. The NZL 5-day and the NZL 10-day sample collections yielded the highest and lowest within-animal variances, respectively (Table 3).

Objectives of this study were to evaluate the effects of extended sample collections (5-day and 10-day), as well as of sample collection apparatuses (ARG *vs.* NZL) on CH_4_ emission estimates by the SF_6_ tracer technique. It was expected that the mean CH_4_ emission estimates would not differ either between the sample collection systems (ARG and NZL) or among the sample collection periods (daily, 5-day and 10-day) within sample collection systems. The above was based on the assumption that the average CH_4_/SF_6_ concentrations in samples would be similar across the sample collection systems and sample collection periods. Overall, the mean CH_4_ emissions estimates of the ARG and NZL systems were only numerically different, whereas when collection systems were compared within sample collection periods, the ARG 5-day collection yielded smaller CH_4_ emission estimates than the NZL 5-day. Reasons for the latter are unknown. If during extended sample collection period the sub-atmospheric internal pressure in the sampling line had favoured slow dilution of the sample by air leaking into the ARG collection canister through the tube connectors, its effect on emission estimates could only be exerted when the CH_4_/SF_6_ ratio of concentrations in the air leaking in, was significantly different from that in the sample inlet.

The NZL system yielded a very high sampling success; hence evaluation of effects of sample collection duration on the mean CH_4_ emission estimates focuses on this system only. Within this system, the three sample collection periods yielded similar CH_4_ emission estimates, with the largest numerical difference of 4.2 g d^−1^ (*i.e.*, 3.8%) observed between NZL daily and NZL 5-day collection periods. Further, when data for the NZL 5-day was split into the corresponding two collection periods (*i.e.*, days 1−5 and days 6−10), their mean CH_4_ emission estimates (108 and 112 g d^−1^, respectively) did not differ (*P* > 0.05) from the estimates based on daily samplings carried out over the same periods (113 and 116 g d^−1^, respectively). Thus, under the controlled conditions of this study, the extended sample collections (5-day and 10-day) proved to yield CH_4_ emission estimates similar to that from the repeated daily sample collections. The high coefficients of correlation among sample collection periods (Table 4) support the consistency of the relationships. Observations within the NZL system suggest that extended sample collection may be an alternative to the daily sampling protocol, whereas the lack of success of the ARG system for a similar outcome may be due to some pitfalls in the sampling process arising from the assembly of the flow restrictor and extended sample line (5 m of a 1/4"o.d. tube), which was imposed by the experimental requirements. 

**Table 4:** Coefficients of correlation (probability value in italics) between mean CH_4_ emission estimates from daily, 5-day and 10-day sample collection periods. Analysis carried out separately for the ARG and NZL sample collection apparatuses.

	ARG system			NZL system		
	Daily (1–10)	5-day (1–5 and 6–10)	10-day (1–10)	Daily (1–10)	5-day (1–5 and 6–10)	10-day (1–10)
Daily	1.00			1.00		
	*0.00*			*0.00*		
5-day	0.67	1.00		0.95	1.00	
	*0.07*	*0.00*		*<0.001*	*0.00*	
10-day	0.86	0.86	1.00	0.98	0.96	1.00
	*0.01*	*0.01*	*0.00*	*<0.001*	*<0.001*	*0.00*

It was also expected from this study that between-animal variances would not differ between sample collection systems (ARG and NZL) or between collection periods, but within-animal variances would decrease with extended sample collection duration. Although sample losses precluded thorough interpretation, it seems that within the NZL system, the between-animal variance of the daily samplings was similar to that of the 10-day samplings. It has been suggested [7] that time-averaging of variation of CH_4_ and SF_6_ mixing ratios at the sampling point (animal’s nostrils) due to micro-meteorological and particular behaviours of tracer and tracee gases would reduce variation of emission estimates when the sampling period is extended. This, however, could not be fully tested in this study as extended sample collections were not repeated enough times; *i.e.*, there were only two values for the 5-day sampling and duplicate samples for the 10-day sampling compared to ten values for the daily sampling. Similarly, comparisons of within-animal variances between similar collection periods (e.g., ARG daily *vs.* NZL daily) were not possible due to the confounding effects of dissimilar sample losses between the systems (ARG *vs.* NZL). 

Studies carried out by Murray *et al*. [13] in sheep found that most (87%) of the enteric CH_4_ production was accounted for by rumen fermentation and that almost all (95%) of the ruminal CH_4_ was eructed, whereas about 89% of the hindgut CH_4_ production was absorbed and excreted through the lungs together with the absorbed ruminal CH_4_. Since the majority (70−99%) of the eructed gases are first inhaled into the lungs, and then expired along with respiratory gases [14,15,16] and provided that eructated and respired gases are well mixed before expiration, the SF_6_ tracer technique should account for about 98% of the total production of CH_4_. Given that the cows in this study had a common fixed feed intake across sampling systems and sampling periods, it was expected that the mean CH_4_ emission estimates should not differ significantly across sampling systems or sampling periods. The results in Table 3 show that mean CH_4_ emissions as measured by the NZL system during daily, 5-day and 10-day collection periods and by the ARG system as measured by daily collection periods, were not different.

In this study, a similar sample source for all the sample collection alternatives (eight in total) was attempted by fitting all the sample inlets within an area of approximately 18 cm^2^ (6 cm × 3 cm) above the nostrils of each animal. The NZL sampling lines had a common sampling area given by the area of the inverted ‘Y’ connector, to which the 1/8" sample lines were attached, whereas each of the ARG sampling lines had an inlet circumference of approx. 1.2" (corresponding to that of the flow restrictor), hence occupying a larger area than the NZL sample inlets. Consequently, the provision of similar or common breath samples to both systems during all collection periods was unlikely to have been attained. Lassey *et al.* [17] suggested that ruminal movements and turbulent eructation processes may thoroughly mix the CH_4_ and SF_6_ gases before they are sampled. However, it is well established that CH_4_ is also exhaled with respiration gases (it is unknown whether SF_6_ is exhaled) and this process seems less turbulent and much less a variable event than eructation. Therefore, exhaled gases may require a mixing action from wind and animal movement (both limited at penned conditions) to warrant a representative sampling. We speculate that the apparent difference in emission estimates between the sample collection periods within the ARG system, may have been due to an uncommon and unrepresentative sampling caused by micrometeorological conditions that were not uniform for all the sampling lines, *i.e.*, because of their larger size, the inlets for the ARG system were co-located in a larger area, whereas the inlets for the NZL system were close within a small area (see Figure 1). Therefore, within the ARG system, effects of sample collection duration and location of sample inlets around the nostrils on CH_4_ emission estimates were likely confounded. 

The SF_6_ tracer technique remains the method of choice for CH_4_ emission estimates from free ranging ruminants. Under such circumstances extended sample collections may be advantageous in terms of their reduced variability of emission estimates ascribed to its time-averaging effect and practicability of its application, especially for grasslands of low carrying capacity. However, extended sample collection protocols may be at higher risk of sample losses than daily samplings. For example, additive action of blocking elements (e.g., dust and water) will occur over an extended period, and so will be the impact of animal behaviour (e.g., chewing of lines, interaction with peers, *etc.*). These potential advantages and problems together with amelioration interventions should be investigated under grazing conditions. 

## 4. Conclusions

This indoor controlled study found that when sample losses were acceptable, the CH_4_ emission estimates based on extended sample collections (5-day or 10-day) did not differ from those based on daily samplings. However, this finding does not necessarily imply that extended sample collection can be used under extensive grazing conditions. Therefore, the extended sampling approach needs to be tested with free-ranging animals before its application can be recommended. Parallel evaluation of advantages (e.g., reduced cost of analyses of samples, reduced labour costs, averaging effect on variability of emissions estimates, animal welfare) and disadvantages (e.g., sample losses) of this approach over the daily sampling protocol are also required. Sample losses and dissimilar CH_4_ emission estimates observed for the system used in Argentina (ARG) most likely were due to experimental conditions; hence a definitive comparison of systems should be carried out under grazing situations. 
